# Manipulating the spectral collapse in two-photon Rabi model

**DOI:** 10.1038/s41598-020-75981-y

**Published:** 2020-10-30

**Authors:** C. F. Lo

**Affiliations:** Department of Physics, Institute of Theoretical Physics, The Chinese University of Hong Kong, Shatin, New Territories Hong Kong

**Keywords:** Physics, Quantum physics

## Abstract

We have investigated the eigenenergy spectrum of the two-photon Rabi model with a full quadratic coupling, particularly the special feature “spectral collapse”. The critical coupling strength is reduced by half from that of the two-photon Rabi model, implying that the spectral collapse can now occur at a more attainable value of the critical coupling. At the critical coupling some discrete eigenenergy levels still survive below the continuous energy spectrum, i.e. an incomplete spectral collapse, and the set of discrete eigenenergies has a one-to-one mapping with that of a particle of variable effective mass in a finite potential well. Since the energy difference between the two atomic levels specifies the depth of the potential well, the number of bound states available (or the extent of the “spectral collapse”) can be straightforwardly monitored. Obviously, this bears a great resemblance to the spectral collapse of the two-photon Rabi model, at least qualitatively. Moreover, since the full quadratic coupling includes an extra term proportional to the photon number operator only, our analysis indicates that one may manipulate the critical coupling of the two-photon Rabi model by incorporating an adjustable proportionality constant to this extra term.

## Introduction

Due to technological advancement in the past decade, the two-photon Rabi model

$$\begin{aligned} H=\omega _{0}S_{z}+\omega a^{\dag }a+2\epsilon \left( a^{\dag 2}+a^{2}\right) S_{x} \end{aligned}$$

has attracted much attention in the literature for its applications are no longer limited to the weak coupling regime^1–11^. Among its various properties, “ spectral collapse” is the most striking feature and occurs when the light-matter interaction coupling strength $\epsilon$ goes beyond a critical value $\epsilon _{c}$. Specifically, above the critical coupling $\epsilon _{c}$ the set of discrete eigenenergy levels of the model turns into a continuous energy spectrum^12–23^. Recently, Lo^24^ has also demonstrated that at the critical coupling $\epsilon _{c}$ some discrete eigenenergy levels still survive below the continuous energy spectrum, i.e. an incomplete spectral collapse, and the set of discrete eigenenergies has a one-to-one mapping with that of a particle in a finite “ Lorentzian function” potential well. Since the energy difference $\omega _{0}$ between the two atomic levels specifies the depth of the potential well, the number of bound states available (or the extent of the “ spectral collapse” ) can be straightforwardly monitored.

Likewise, Felicetti et al.^4^ have demonstrated via numerical calculations that replacing the term $a^{\dag 2}+a^{2}$ in the light-matter interaction by a full quadratic term $\left( a^{\dag }+a\right) ^{2}$ reduces the critical coupling strength by a factor of two. That is, the spectral collapse occurs at a more attainable value of the critical coupling. The full quadratic coupling is indeed the actual physical representation of the light-matter interaction for the two-level atomic system (or qubit) is coupled to the square of the electric or magnetic field. They also argue that the addition of more qubits further lowers the critical value of the individual qubit coupling by a factor of *N*, where *N* denotes the number of qubits present. If their theoretical proposals can be related to feasible experiments, such a scaling of the critical coupling with the number of qubits may enable us to experimentally achieve the critical coupling strength to yield the spectral collapse with the state-of-the-art circuit quantum electrodynamics technology. Nevertheless, our understanding of the spectral collapse occuring in the two-photon Rabi model with a full quadratic coupling is very limited because current theoretical approaches (both analytical and numerical) fail in dealing with the collapse point rigorously. In particular, whether the spectral collapse of such a model is different from that of the two-photon Rabi model remains as a mystery.

Accordingly, it is the aim of our work to delve into this mystery. The critical value of the coupling strength of the two-photon Rabi model with a full quadratic coupling is found to be reduced by half from that of the two-photon Rabi model. At the critical coupling the discrete eigenenergy spectrum of the incomplete spectral collapse can be derived via a simple one-to-one mapping to the bound state problem of a particle of variable effective mass in a finite potential well. This bears a great resemblance to that of the two-photon Rabi model, at least qualitatively. Moreover, our analysis indicates that one may manipulate the critical coupling of the two-photon Rabi model by incorporating an extra coupling term $4\chi \epsilon \left( a^{\dag }a+\frac{1}{2}\right) S_{x}$ into its Hamiltonian, where $\chi$ is an adjustable positive parameter, and that the resultant critical coupling is given by

$$\begin{aligned} \epsilon _{c}=\frac{\omega }{2\left( 1+\chi \right) }. \end{aligned}$$

## Two-photon Rabi model with the full quadratic coupling

The two-photon Rabi model with a full quadratic coupling is described by the Hamiltonian $\left( \hbar =1\right)$^4^:

1 $$\begin{aligned} H=\omega _{0}S_{z}+\omega a^{\dag }a+2\epsilon \left( a^{\dag }+a\right) ^{2}S_{x}\ . \end{aligned}$$

The various coupling regimes of the model can be specified in terms of the three model parameters, namely the frequency $\omega$ of the radiation mode, the energy difference $\omega _{0}$ between the two atomic levels, and the coupling strength $\epsilon$ of the light-matter interaction. It is obvious that this model differs from the two-photon Rabi model by the presence of an extra coupling term $4\epsilon \left( a^{\dag }a+\frac{1}{2} \right) S_{x}$ in its Hamiltonian only. Without loss of generality, we set the energy unit such that $\omega =1$ for simplicity in the following analysis.

To begin with, we define the “ position” and “ momentum” operators of the boson mode as

2 $$\begin{aligned} x=\frac{1}{\sqrt{2}}\left( a+a^{\dag }\right) \qquad \text {and}\qquad p= \frac{1}{i\sqrt{2}}\left( a-a^{\dag }\right) , \end{aligned}$$

respectively. Then the Hamiltonian *H* can be expressed as

3 $$\begin{aligned} H=\omega _{0}S_{z}+\frac{p^{2}+x^{2}}{2}+4\epsilon x^{2}S_{x}-\frac{1}{2}\ . \end{aligned}$$

In the special case of $\omega _{0}=0$ the Hamiltonian *H* is reduced to

4 $$\begin{aligned} H=\frac{p^{2}}{2}+\frac{x^{2}}{2}\left( 1+8\epsilon S_{x}\right) -\frac{1}{2} \ , \end{aligned}$$

and its eigenstates are simply given by the states $\left\{ |M_{x}\rangle |\phi \rangle \right\}$, where $|M_{x}\rangle$ is an eigenstate of the spin operator $S_{x}$ and $|\phi \rangle$ is an eigenstate of the one-body Hamiltonian *h*:

5 $$\begin{aligned} h=\left\{ \begin{array}{c} \frac{1}{2}p^{2}+\frac{1}{2}\left( 1-4\epsilon \right) x^{2}-\frac{1}{2} \quad \text {for\ }M_{x}=-\frac{1}{2} \\ \frac{1}{2}p^{2}+\frac{1}{2}\left( 1+4\epsilon \right) x^{2}-\frac{1}{2} \quad \text {for\ }M_{x}=\frac{1}{2} \end{array} \right. \ . \end{aligned}$$

It is obvious that in the subspace of $M_{x}=\frac{1}{2}$ the one-body Hamiltonian *h* describes a quantum simple harmonic oscillator for all $\epsilon >0$. On the other hand, there exists a critical value of the coupling strength, namely $\epsilon _{c}\equiv \frac{1}{4}$, in the subspace of $M_{x}=-\frac{1}{2}$. For $\epsilon <\epsilon _{c}$ the one-body Hamiltonian *h* corresponds to a quantum simple harmonic oscillator, whereas it represents an inverted harmonic potential barrier for $\epsilon >\epsilon _{c}$. In addition, at the critical coupling $\epsilon _{c}$ the system behaves like a free particle. This abrupt change in the fundamental nature of the system is responsible for the transformation from a discrete eigenenergy spectrum to a continuous energy spectrum. For $\omega _{0}\ne 0$ the two subspaces no longer exist since the spin degree of freedom and the boson mode cannot be decoupled. When the two subspaces are mixed, the above analysis of the existence of a critical coupling still holds because the first term in Eq. (3) is a bounded operator. Nevertheless, the characteristic behaviour of the eigenstates at the critical coupling $\epsilon _{c}$ remains as a mystery.

Moreover, as shown in Ng et al.^12^, the unitary transformation

6 $$\begin{aligned} R=\exp \left\{ -\frac{i\pi }{2}\left( S_{x}-\frac{1}{2}\right) a^{\dag }a\right\} \end{aligned}$$

may be applied to transform the Hamiltonian *H* in Eq. (1) to

7 $$\begin{aligned} {\bar{H}}\,=\, & {} R^{\dag }HR \nonumber \\ \,=\, & {} \omega _{0}\cos \left( \frac{\pi }{2}a^{\dag }a\right) S_{z}+\omega _{0}\sin \left( \frac{\pi }{2}a^{\dag }a\right) S_{y}+\left( a^{\dag }a+ \frac{1}{2}\right) \left( 1+4\epsilon S_{x}\right) + \nonumber \\&\epsilon \left( a^{\dag 2}+a^{2}\right) -\frac{1}{2}\ . \end{aligned}$$

At the critical coupling $\epsilon _{c}\equiv 1/4$, the Hamiltonian ${\bar{H}}$ becomes

8 $$\begin{aligned} {\bar{H}}_{even}=\omega _{0}\cos \left( \frac{\pi }{2}a^{\dag }a\right) S_{z}+\left( a^{\dag }a+\frac{1}{2}\right) \left( 1+S_{x}\right) +\epsilon \left( a^{\dag 2}+a^{2}\right) -\frac{1}{2} \end{aligned}$$

in the subspace of even number states of $a^{\dag }a$ whereas we have

9 $$\begin{aligned} {\bar{H}}_{odd}=\omega _{0}\sin \left( \frac{\pi }{2}a^{\dag }a\right) S_{y}+\left( a^{\dag }a+\frac{1}{2}\right) \left( 1+S_{x}\right) +\epsilon \left( a^{\dag 2}+a^{2}\right) -\frac{1}{2} \end{aligned}$$

in the subspace of odd number states. Contrary to the two-photon Rabi model, it is apparent that in both cases the spin degree of freedom and the boson mode cannot be decoupled. The two-fold degeneracy of each eigenenergy of the two-photon Rabi model corresponding to the spin degree of freedom^24^ has thus been lifted by the presence of the extra term $\left( a^{\dag }a+\frac{1}{2}\right) S_{x}$ in the Hamiltonian ${\bar{H}}$. Consequently, the eigenstates and eigenenergies of *H* are manifestly different from those of the two-photon Rabi model.

## Eigenenergy spectrum at the critical coupling

To derive the eigenenergy spectrum of *H* at the critical coupling $\epsilon _{c}\equiv \frac{1}{4}$, we first perform a spin rotation

10 $$\begin{aligned} T=\exp \left\{ -\frac{i\pi }{2}S_{y}\right\} \end{aligned}$$

to Eq.(3) and obtain

11 $$\begin{aligned} H=\omega _{0}S_{x}+\frac{p^{2}+x^{2}}{2}-x^{2}S_{z}-\frac{1}{2}\ , \end{aligned}$$

whose eigenvalue equation in the momentum space reads

12 $$\begin{aligned} E\left( \begin{array}{c} {\tilde{\psi }}_{+}\left( p\right) \\ {\tilde{\psi }}_{-}\left( p\right) \end{array} \right)= & {} \left( \begin{array}{cc} \frac{1}{2}p^{2}-\frac{1}{2} &{} \frac{1}{2}\omega _{0} \\ \frac{1}{2}\omega _{0} &{} -\frac{d^{2}}{dp^{2}}+\frac{1}{2}p^{2}-\frac{1}{2} \end{array} \right) \left( \begin{array}{c} {\tilde{\psi }}_{+}\left( p\right) \\ {\tilde{\psi }}_{-}\left( p\right) \end{array} \right) \nonumber \\= & {} \left( \begin{array}{c} \left( \frac{1}{2}p^{2}-\frac{1}{2}\right) {\tilde{\psi }}_{+}\left( p\right) + \frac{1}{2}\omega _{0}{\tilde{\psi }}_{-}\left( p\right) \\ \frac{1}{2}\omega _{0}{\tilde{\psi }}_{+}\left( p\right) +\left( -\frac{d^{2}}{ dp^{2}}+\frac{1}{2}p^{2}-\frac{1}{2}\right) {\tilde{\psi }}_{-}\left( p\right) \end{array} \right) \ . \end{aligned}$$

Here *E* denotes the energy of the eigenstate $$\left( \begin{array}{c} {\tilde{\psi }}_{+}\left( p\right) \\ {\tilde{\psi }}_{-}\left( p\right) \end{array} \right)$$. From Eq. (12) we can easily derive

13 $$\begin{aligned} {\tilde{\psi }}_{+}\left( p\right) =\frac{\omega _{0}}{2E+1-p^{2}}{\tilde{\psi }} _{-}\left( p\right) \end{aligned}$$

and

14 $$\begin{aligned} \left( -\frac{d^{2}}{dp^{2}}+\frac{1}{2}p^{2}-\frac{1}{2}\right) {\tilde{\psi }} _{-}\left( p\right) +\frac{1}{2}\omega _{0}{\tilde{\psi }}_{+}\left( p\right) =E {\tilde{\psi }}_{-}\left( p\right) \ . \end{aligned}$$

Substituting Eq. (13) into Eq. (14) then yields

15 $$\begin{aligned} -2\frac{d^{2}{\tilde{\psi }}_{-}\left( p\right) }{dp^{2}}+\frac{\omega _{0}^{2} }{2E+1-p^{2}}{\tilde{\psi }}_{-}\left( p\right) =\left( 2E+1-p^{2}\right) {\tilde{\psi }}_{-}\left( p\right) \ . \end{aligned}$$

For $E+\frac{1}{2}<0$, we introduce the parameter $\kappa =\sqrt{\left| E+\frac{1}{2}\right| }$ and define a new variable $q=\frac{p}{\sqrt{2} \kappa }$ such that Eq. (15) can be rewritten as

16 $$\begin{aligned} -\frac{1}{2\left( 1+q^{2}\right) }\frac{d^{2}{\tilde{\psi }}_{-}\left( q\right) }{dq^{2}}-\frac{\omega _{0}^{2}}{4\left( 1+q^{2}\right) ^{2}}{\tilde{\psi }} _{-}\left( q\right) =-\kappa ^{4}{\tilde{\psi }}_{-}\left( q\right) \ . \end{aligned}$$

Assuming that ${\tilde{\psi }}_{-}\left( q\right)$ takes the form

17 $$\begin{aligned} {\tilde{\psi }}_{-}\left( q\right) =\frac{1}{\sqrt{1+q^{2}}}\phi \left( q\right) \ , \end{aligned}$$

we can easily show that $\phi \left( q\right)$ satisfies

18 $$\begin{aligned} -\frac{1}{2}\left\{ \frac{1}{\sqrt{M\left( q\right) }}\frac{d^{2}}{dq^{2}} \frac{1}{\sqrt{M\left( q\right) }}\right\} \phi \left( q\right) +V\left( q\right) \phi \left( q\right) =-\kappa ^{4}\phi \left( q\right) \ , \end{aligned}$$

which is the time-independent Schrödinger equation of the bound state problem associated with a particle of variable effective^25,26^

19 $$\begin{aligned} M\left( q\right) =1+q^{2} \end{aligned}$$

moving in a finite potential well:

20 $$\begin{aligned} V\left( q\right) =-\frac{\omega _{0}^{2}}{4\left( 1+q^{2}\right) ^{2}}\ . \end{aligned}$$

In order to faciliate a better understanding of the eigenenergy spectrum, we introduce the unitary transformation

21 $$\begin{aligned} U=\exp \left\{ \frac{i}{2}\left[ \left( \frac{1}{i}\frac{d}{dq}\right) f\left( q\right) +f\left( q\right) \left( \frac{1}{i}\frac{d}{dq}\right) \right] \right\} \ , \end{aligned}$$

for some function $f\left( q\right)$. It is not difficult to show that *U* transforms *q* and $\frac{1}{i}\frac{d}{dq}$ as follows:

22 $$\begin{aligned} U^{\dag }qU= & {} q+F\left( q\right) \end{aligned}$$

23 $$\begin{aligned} U^{\dag }\left( \frac{1}{i}\frac{d}{dq}\right) U= & {} \frac{1}{2}\left\{ \left( \frac{1}{i}\frac{d}{dq}\right) G\left( q\right) +G\left( q\right) \left( \frac{1}{i}\frac{d}{dq}\right) \right\} \ , \end{aligned}$$

where

24 $$\begin{aligned} F\left( q\right)= & {} \sum _{n=1}^{\infty }\frac{\left( -1\right) ^{n}}{n!} f_{n}\left( q\right) \quad ,\quad f_{n+1}\left( q\right) =f\left( q\right) \frac{df_{n}\left( q\right) }{dq}\quad ,\quad f_{1}\left( q\right) =f\left( q\right) \end{aligned}$$

25 $$\begin{aligned} G\left( q\right)= & {} \sum _{n=0}^{\infty }\frac{\left( -1\right) ^{n}}{n!} g_{n}\left( q\right) \quad ,\quad g_{n+1}\left( q\right) =f^{2}\left( q\right) \frac{d}{dq}\left\{ \frac{g_{n}\left( q\right) }{f\left( q\right) } \right\} \quad ,\quad g_{0}\left( q\right) =1\ . \end{aligned}$$

Obviously, we must require

26 $$\begin{aligned} \frac{d}{dq}\left\{ q+F\left( q\right) \right\} =\frac{1}{G\left( q\right) } \end{aligned}$$

in order that $\left[ U^{\dag }qU,U^{\dag }\left( \frac{1}{i}\frac{d}{dq} \right) U\right] =\left[ q,\frac{1}{i}\frac{d}{dq}\right] =i$. That is, the commutation relation between *q* and $\frac{1}{i}\frac{d}{dq}$ are preserved under the unitary transformation *U*. Then, applying the unitary transformation *U* to Eq. (18) gives

27 $$\begin{aligned} -\frac{1}{2}\left\{ \frac{G\left( q\right) }{\sqrt{M\left( q+F\left( q\right) \right) }}\frac{d^{2}}{dq^{2}}\frac{G\left( q\right) }{\sqrt{ M\left( q+F\left( q\right) \right) }}\right\} {\bar{\phi }}\left( q\right) + {\bar{V}}\left( q\right) {\bar{\phi }}\left( q\right) =-\kappa ^{4}{\bar{\phi }} \left( q\right) \ , \end{aligned}$$

where $${\bar{\phi }}\left( q\right) =U^{\dag }\phi \left( q\right)$$ and

28 $$\begin{aligned} {\bar{V}}\left( q\right) =V\left( q+F\left( q\right) \right) +\frac{1}{ 8M\left( q+F\left( q\right) \right) }\left\{ \frac{d^{2}G^{2}\left( q\right) }{dq^{2}}-3\left[ \frac{dG\left( q\right) }{dq}\right] ^{2}\right\} \ . \end{aligned}$$

By setting

29 $$\begin{aligned} G\left( q\right) =\sqrt{1+\left[ q+F\left( q\right) \right] ^{2}}\ , \end{aligned}$$

Equation (27) is reduced to

30 $$\begin{aligned} -\frac{1}{2}\frac{d^{2}{\bar{\phi }}\left( q\right) }{dq^{2}}+{\bar{V}}\left( q\right) {\bar{\phi }}\left( q\right) =-\kappa ^{4}{\bar{\phi }}\left( q\right) \ , \end{aligned}$$

where

31 $$\begin{aligned} {\bar{V}}\left( q\right) =-\frac{2\omega _{0}^{2}+3}{8\left\{ 1+\left[ q+F\left( q\right) \right] ^{2}\right\} ^{3}}\left\{ \left[ q+F\left( q\right) \right] ^{2}+\frac{\omega _{0}^{2}-1}{\omega _{0}^{2}+\frac{3}{2}} \right\} \ . \end{aligned}$$

Provided that $\omega _{0}>1$, ${\bar{V}}\left( q\right)$ is negative definite and represents a finite potential well. On the other hand, if $\omega _{0}<1$, then we have

32 $$\begin{aligned} \left\{ \begin{array}{cc} {\bar{V}}\left( q\right) >0 &{} \quad \text {for}\quad \left[ q+F\left( q\right) \right] ^{2}<\left( 1-\omega _{0}^{2}\right) /\left( \omega _{0}^{2}+\frac{3 }{2}\right) \\ {\bar{V}}\left( q\right) <0 &{}\quad \text {otherwise} \end{array} \right. \ . \end{aligned}$$

In addition, Eq. (26) yields

33 $$\begin{aligned} q+F\left( q\right) =\sinh \left( 2q-\left[ q+F\left( q\right) \right] \sqrt{ 1+\left[ q+F\left( q\right) \right] ^{2}}\right) \ , \end{aligned}$$

from which $F\left( q\right)$ can be determined. Consequently, we have succeeded in transforming Eq. (18) into the time-independent Schrödinger equation of the bound state problem associated with a particle of unit mass moving in the finite potential well ${\bar{V}}\left( q\right)$, and this system has a set of discrete bound state eigenenergy levels.

For $E+1/2>0$, in terms of the parameter $k=\sqrt{E+1/2}$ and the new variable ${\bar{q}}=p/\left( \sqrt{2}k\right)$, Eq. (15) becomes

34 $$\begin{aligned} -\frac{1}{2\left( 1-{\bar{q}}^{2}\right) }\frac{d^{2}{\tilde{\psi }}_{-}\left( {\bar{q}}\right) }{d{\bar{q}}^{2}}+\frac{\omega _{0}^{2}}{4\left( 1-{\bar{q}} ^{2}\right) ^{2}}{\tilde{\psi }}_{-}\left( {\bar{q}}\right) =k^{4}{\tilde{\psi }} _{-}\left( {\bar{q}}\right) \ . \end{aligned}$$

Again, if ${\tilde{\psi }}_{-}\left( {\bar{q}}\right)$ assumes the form

35 $$\begin{aligned} {\tilde{\psi }}_{-}\left( {\bar{q}}\right) =\frac{1}{\sqrt{1-{\bar{q}}^{2}}}\phi \left( {\bar{q}}\right) \ , \end{aligned}$$

then $$\phi \left( {\bar{q}}\right)$$ obeys

36 $$\begin{aligned} -\frac{1}{2}\left\{ \frac{1}{\sqrt{M\left( {\bar{q}}\right) }}\frac{d^{2}}{d {\bar{q}}^{2}}\frac{1}{\sqrt{M\left( {\bar{q}}\right) }}\right\} \phi \left( {\bar{q}}\right) +V\left( {\bar{q}}\right) \phi \left( {\bar{q}}\right) =k^{4}\phi \left( {\bar{q}}\right) \end{aligned}$$

which is the time-independent Schrödinger equation of the scattering state problem associated with a particle of variable effective mass^25,26^

37 $$\begin{aligned} M\left( {\bar{q}}\right) =1-{\bar{q}}^{2} \end{aligned}$$

and the potential barrier:

38 $$\begin{aligned} V\left( {\bar{q}}\right) =\frac{\omega _{0}^{2}}{4\left( 1-{\bar{q}}^{2}\right) ^{2}}\ . \end{aligned}$$

Here both $M^{-1}\left( {\bar{q}}\right)$ and $V\left( {\bar{q}}\right)$ are singular at ${\bar{q}}=\pm 1$.

In summary, the critical value of the coupling strength has been reduced by half from that of the two-photon Rabi model. At the critical coupling $\epsilon _{c}$ the system not only has a set of discrete eigenenergies but also has a continuous energy spectrum. In Eq. (20) the parameter $\omega _{0}$ specifies the depth of the finite potential well and determines the number of bound states available. On the other hand, in Eq. (38) the parameter $\omega _{0}$ specifies the magnitude of the potential barrier. Moreover, unlike the two-photon Rabi model in which each eigenstate is doubly degenerate due to the spin degree of freedom, the two-fold degeneracy has been lifted by the presence of the extra term $4\epsilon \left( a^{\dag }a+\frac{1}{2}\right) S_{x}$ in the Hamiltonian *H* of the two-photon Rabi model with a full quadratic coupling, as shown in Eq. (1).

## Discussion and conclusion

In this communication we have rigorously shown that replacing the term $a^{\dag 2}+a^{2}$ in the light-matter interaction of the two-photon Rabi model by a full quadratic term $\left( a^{\dag }+a\right) ^{2}$ reduces the critical coupling strength by half. Thus, the spectral collapse can now occur at a more attainable value of the critical coupling. At the critical coupling $\epsilon _{c}\equiv 1/4$ the eigenenergy spectrum of the two-photon Rabi model with a full quadratic coupling consists of both a set of discrete energy levels and a continuous energy spectrum. The discrete eigenenergy spectrum has a one-to-one mapping with that of a particle of variable effective mass in a finite potential well, and the continuous energy spectrum can be derived from the scattering problem associated with a potential barrier. Despite its simplicity, we need to resort to numerical methods to determine the eigenenergies and eigenstates explicitly.

In short, the two-photon Rabi model with a full quadratic coupling has three different regimes: $\left( 1\right)$ a purely discrete eigenenergy spectrum for $\epsilon <\epsilon _{c}$, $\left( 2\right)$ a purely continuous energy spectrum for $\epsilon >\epsilon _{c}$, and $\left( 3\right)$ a combination of a set of discrete energy levels and a continuous energy spectrum at $\epsilon =\epsilon _{c}$. The number of bound states available at the critical coupling $\epsilon _{c}$ can be controlled by adjusting the parameter $\omega _{0}$, implying that the extent of the spectral collapse can be monitored in a straightforward manner. It is obvious that this bears a qualitative resemblance to the spectral collapse of the two-photon Rabi model. Nevertheless, there exist some significant quantitative discrepancies; for instance, the two-fold degeneracy of each eigenstate of the two-photon Rabi model associated with the spin degree of freedom has been lifted.

Finally, as implied by our analysis, one may manipulate the critical coupling of the two-photon Rabi model by incorporating an extra coupling term $4\chi \epsilon \left( a^{\dag }a+\frac{1}{2}\right) S_{x}$ into its Hamiltonian $\left( \hbar =1\right)$:

39 $$\begin{aligned} H \,=\, & {} \omega _{0}S_{z}+\omega a^{\dag }a+2\epsilon \left( a^{\dag 2}+a^{2}\right) S_{x}+4\chi \epsilon \left( a^{\dag }a+\frac{1}{2}\right) S_{x} \nonumber \\ \,=\, & {} \omega _{0}S_{z}+\omega a^{\dag }a+2\epsilon \left( a^{\dag }+a\right) ^{2}S_{x}+4\left( \chi -1\right) \epsilon \left( a^{\dag }a+\frac{1}{2} \right) S_{x}\ , \end{aligned}$$

where $\chi$ is an adjustable positive parameter^27^. For $\chi =1$, we recover the two-photon Rabi model with a full quadratic coupling. The resultant critical coupling can be straightforwardly shown to be

40 $$\begin{aligned} \epsilon _{c}=\frac{\omega }{2\left( 1+\chi \right) }\ . \end{aligned}$$

Thus, an increase in the value of the parameter $\chi$ diminishes the critical value of the coupling strength, suggesting that this may enable us to experimentally achieve the critical coupling strength to yield the spectral collapse with the state-of-the-art circuit quantum electrodynamics technology. The eigenvalue equation of *H* corresponding to Eq. (12) is given by $\left( \omega =1\right)$

41 $$\begin{aligned} E\left( \begin{array}{c} {\tilde{\psi }}_{+}\left( p\right) \\ {\tilde{\psi }}_{-}\left( p\right) \end{array} \right) =\left( \begin{array}{c} \left( \frac{1}{1+\chi }p^{2}-\frac{1}{2}\right) {\tilde{\psi }}_{+}\left( p\right) +\frac{1}{2}\omega _{0}{\tilde{\psi }}_{-}\left( p\right) \\ \frac{1}{2}\omega _{0}{\tilde{\psi }}_{+}\left( p\right) +\left( -\frac{d^{2}}{ dp^{2}}+\frac{\chi }{1+\chi }p^{2}-\frac{1}{2}\right) {\tilde{\psi }}_{-}\left( p\right) \end{array} \right) \ , \end{aligned}$$

from which we can obtain

42 $$\begin{aligned} {\tilde{\psi }}_{+}\left( p\right) =\frac{\omega _{0}}{2E+1-\frac{2}{1+\chi } p^{2}}{\tilde{\psi }}_{-}\left( p\right) \end{aligned}$$

and

43 $$\begin{aligned} -2\frac{d^{2}{\tilde{\psi }}_{-}\left( p\right) }{dp^{2}}+\frac{\omega _{0}^{2} }{2E+1-\frac{2}{1+\chi }p^{2}}{\tilde{\psi }}_{-}\left( p\right) =\left( 2E+1- \frac{2\chi }{1+\chi }p^{2}\right) {\tilde{\psi }}_{-}\left( p\right) \ . \end{aligned}$$

Obviously, for $\chi =1$, Eqs. (42) and (43) are reduced to Eqs. (13) and (15), respectively. As a result, we can apply the same procedures shown in Sect. 3 to derive that at the critical coupling the eigenenergy spectrum of this system consists of both a set of discrete eigenenergies and a continuous energy spectrum.
